# A Statistical Framework for Measuring Reproducibility and Replicability of High‐Throughput Experiments From Multiple Sources

**DOI:** 10.1002/sim.70354

**Published:** 2026-02-04

**Authors:** Monia Ranalli, Yafei Lyu, Hillary Koch, Qunhua Li

**Affiliations:** ^1^ Department of Statistics Sapienza University of Rome Rome Italy; ^2^ Bioinformatics and Genomics Graduate Program, The Huck Institute of the Life Science Pennsylvania State University University Park Pennsylvania USA; ^3^ Department of Statistics Pennsylvania State University University Park Pennsylvania USA

**Keywords:** copula, high‐throughput experiment, irreproducible discovery rate, mixture models, replicability, reproducibility

## Abstract

Replication is essential to reliable and consistent scientific discovery in high‐throughput experiments. Quantifying the replicability of scientific discoveries and identifying sources of irreproducibility have become important tasks for quality control and data integration. In this work we introduce a novel statistical model to measure the reproducibility and replicability of findings from replicate experiments in multi‐source studies. Using a nested copula mixture model that characterizes the interdependence between replication experiments both across and within sources, our method quantifies reproducibility and replicability of each candidate simultaneously in a coherent framework. Through simulation studies, an ENCODE ChIP‐seq dataset and a SEQC RNA‐seq dataset, we demonstrate the effectiveness of our method in diagnosing the source of discordance and improving the reliability of scientific discoveries.

## 1 Introduction

High‐throughput technologies have revolutionized modern biological research by enabling the simultaneous measurement of thousands of molecular candidates in a single experiment. These technologies have facilitated the discovery of key genomic features, such as transcription factor binding sites, differentially expressed genes, and regulatory elements. Over the past decade, large volumes of high‐throughput data have been generated by individual labs and multi‐institutional consortia, such as ENCODE [1, 2] and Roadmap Epigenomics [3]. Many of these datasets measure the same biological features but originate from different labs or different studies, presenting both opportunities and challenges for data integration.

Integrating datasets across sources can improve statistical power and reliability, but is complicated by source‐specific heterogeneity from differences in laboratory conditions, reagent batches, personnel, and protocols [4]. Batch‐correction methods such as ComBat [5] and SVA [6] are often applied to reduce such variation. While effective for removing systematic shifts, these methods alter the original measurements and do not quantify the consistency of findings across and within sources–information that can strengthen confidence in discoveries and is the focus here.

Two concepts central to evaluating consistency in multi‐source studies are replicability and reproducibility [7, 8, 9, 10]. While sometimes used interchangeably, these terms have distinct statistical meanings in the context of high‐throughput data. Reproducibility refers to the consistency of results within a lab across repeated experiments using the same experimental and analysis pipeline, whereas replicability refers to the consistency of findings across independent labs or studies, often involving variations in personnel or procedures. We refer to repeated experiments within a lab as replicates. A signal consistently detected across replicates is deemed reproducible, whereas a signal consistently observed across independent labs is considered replicable.

**FIGURE 1:**
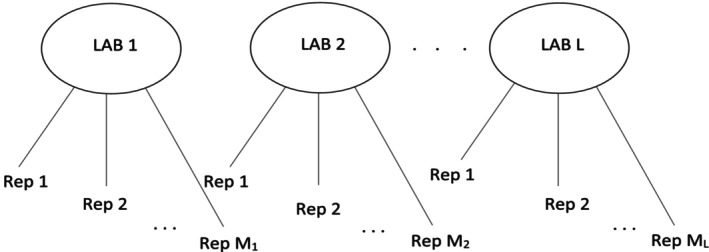
Typical experimental design in consortium studies.

In many high‐throughput multi‐source studies, experiments follow a nested structure (Figure 1): replicates are grouped within labs, and labs function as independent units. Measurements from replicates within the same lab tend to be more consistent than those across labs due to shared experimental conditions. Disentangling reproducibility and replicability in this nested design is essential for identifying the origin of discrepancies and for reliable data integration.

Several statistical methods have been proposed to quantify consistency in multi‐source studies, though they target different inferential goals. The r‐value [11] and the reproducibility discovery rate (RDR) [12, 13] provide global measures of replication: the r‐value is designed for asymmetric two‐stage studies involving pre‐selected findings, whereas RDR summarizes replication rates at the study level. Both offer useful summary metrics, but neither produces candidate‐level, threshold‐independent scores in a symmetric multi‐source setting.

The irreproducible discovery rate (IDR) framework [14] directly models consistency across replicates, estimating the probability that a candidate is irreproducible and boosting confidence in signals supported by multiple replicates. IDR is widely used for ChIP‐seq peak assessment [15, 16] and has also been adapted for cross‐platform gene‐expression analysis [17]. However, it treats all replicates as exchangeable, ignoring the distinction between within‐lab reproducibility and between‐lab replicability and failing to attribute inconsistency to specific sources. Other approaches integrate evidence without explicitly modeling reproducibility, such as rank‐based [18] and *p*‐value based meta‐analysis methods [19, 20]. These approaches favor candidates with consistent evidence, but, like IDR, they treat all replicates as exchangeable and cannot disentangle within‐lab from between‐lab consistency.

Here we introduce nestedIDR, a nested copula mixture model that extends IDR to multi‐lab studies. By modeling the hierarchical dependence structure within each lab and between labs in a single probabilistic framework, nestedIDR captures source‐specific heterogeneity that standard IDR methods overlook. Like IDR, it operates on ranks, ensuring robustness to scale differences without altering the original measurements. The method produces candidate‐level scores for both reproducibility and replicability, enabling investigators to prioritize findings and diagnose source of discordance.

Section 2 describes the nestedIDR model and estimation procedure. Section 3 uses extensive simulation studies to compare nestedIDR with standard IDR and meta‐analysis approaches. Section 4 applies nestedIDR to an ENCODE ChIP‐seq [1, 21] and SEQC RNA‐seq data [22], demonstrating practical gains in power and biological relevance. We conclude in Section 5 with a discussion.

## 2 Method

### 2.1 The NestedIDR Model

Suppose L labs perform independent replications, each with $M_{l}$ replicates for lab l,(l∈{1,…,L}). For each replicate, n candidates are measured using a high‐throughput experiment, resulting in a quantitative score for each candidate (e.g., gene expression levels from RNA‐seq or p‐values from ChIP‐seq) that reflects the strength of evidence supporting the candidate as a genuine signal. We represent the full data as an n×P matrix X, where $P=\sum_{l=1}^{L}M_{l}$ is the total number of replicates. The measurement vector for candidate i is denoted $x_{i}=(x_{i}^{\left(1\right)},\ldots ,x_{i}^{\left(L\right)})$, where $x_{i}^{\left(l\right)}=(x_{i,1}^{\left(l\right)},\ldots ,x_{i,M_{l}}^{\left(l\right)})$ contains measurements from lab l. We denote the marginal distribution of replicate m $\left(m\in \{1,\ldots ,M_{l}\}\right)$ in lab l as $F_{m}^{\left(l\right)}$, which may differ across replicates and labs. Our model only requires the ranks of the values on each replicate; thus, if only rank data are available, $x_{i,m}^{\left(l\right)}$ denotes the rank of candidate i and $F_{m}^{\left(l\right)}$ is its empirical distribution. Without loss of generality, we assume that stronger candidates receive higher ranks.

**FIGURE 2:**
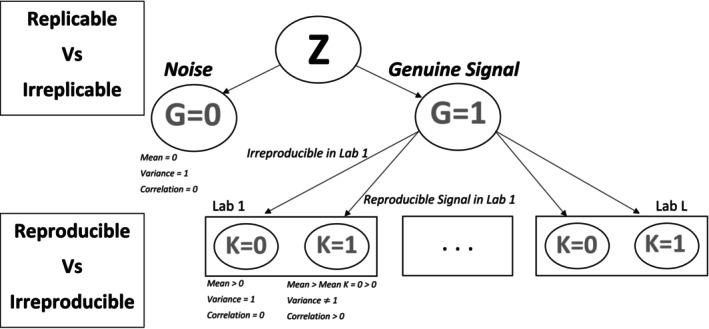
Structure of our model.

Figure 2 illustrates the nested structure motivating our model. In multi‐source experimental designs, replicates are naturally grouped within labs, and labs differ in variability due to protocol, personnel, or environmental factors. It is therefore important to jointly model within‐lab reproducibility and between‐lab replicability in a way that allows for lab‐specific heterogeneity. To achieve this, we treat the observed data as arising from a two‐level stochastic process: the first level models lab‐specific reproducibility, and the second level captures signal replicability across labs. This nested formulation allows us to decouple source‐specific noise from signal structure and appropriately account for heterogeneity in multi‐lab studies.

We assume that each candidate is either a genuine signal ($G_{i}=1$) or noise ($G_{i}=0$), with prior probabilities $\pi_{1}$ and $\pi_{0}=1-\pi_{1}$. Whether a signal is genuine or not is reflected by how well it is replicated across labs. Furthermore, conditional on $G_{i}=1$, the reproducibility in lab l may vary with lab‐specific data quality. For instance, in a ChIP‐seq study, a protein‐binding site may be consistently detected in both replicates of one lab due to sufficient sequencing depth, but fail to be reproducible in another lab because one of its replicates has insufficient coverage. Such replicate‐specific variation motivates the need to model reproducibility at the lab level. Let $K_{i}^{\left(l\right)}\in \{0,1\}$ indicate whether a genuine signal is reproducible in lab l ($K_{i}^{\left(l\right)}=1$) or not ($K_{i}^{\left(l\right)}=0$), with probabilities $\pi_{1|1}^{\left(l\right)}$ and $\pi_{0|1}^{\left(l\right)}=1-\pi_{1|1}^{\left(l\right)}$. For $G_{i}=0$, we set $K_{i}^{\left(l\right)}=0$ for all l, reflecting that noise is not reproducible. Note that while nestedIDR assumes conditional independence of lab‐specific measurements given $G_{i}$, the shared $G_{i}$ induces marginal dependence across labs. This structure allows all replicates‐from all sources‐to contribute jointly to the posterior replicability score, borrowing strength across labs while explicitly modeling source‐specific heterogeneity.

To model the hierarchical dependence structure while remaining robust to differences in measurement scales across labs and replicates, we extend the semiparametric copula framework in IDR [14]. The copula captures dependence across replicates parametrically, while the marginal distributions are estimated nonparametrically from ranks. This permits arbitrary measurement scales and enables robust inference in the presence of heterogeneous distributions.

Let $z_{i}^{\left(l\right)}={\left(z_{i,1}^{\left(l\right)},\ldots ,z_{i,M_{l}}^{\left(l\right)}\right)}^{T}$ denote the latent vector for lab l. We assume independence across labs, that is, $z_{i}^{\left(l\right)}$ and $z_{i}^{\left(l^{\prime }\right)}$ are independent for $l\neq l^{\prime }$, to reflect the common experimental design where labs operate independently. We model

(1) $$z_{i}^{\left(l\right)}|G_{i}=g,K_{i}^{\left(l\right)}=k∼𝒩(\mu_{k|g}^{\left(l\right)},\sum_{k|g}^{\left(l\right)}),\;\text{for}\;g,k\in \{0,1\}.$$

For parsimony, $\mu_{k|g}^{\left(l\right)}=\mu_{k|g}^{\left(l\right)}1$ and

$$\sum_{k|g}^{\left(l\right)}={\left(\sigma_{k|g}^{\left(l\right)}\right)}^{2}\left((1-\rho_{k|g}^{\left(l\right)})I+\rho_{k|g}^{\left(l\right)}J\right),$$

where I is the identity matrix, J is a matrix of ones, $\sigma_{k|g}^{\left(l\right)}$ is the standard deviation, and $\rho_{k|g}^{\left(l\right)}$ is the intra‐lab correlation. This exchangeable structure reflects the assumption that replicates from the same lab tend to share similar quality and technical variation, and can be readily modified to accommodate non‐exchangeable structures.

We model noise ($G_{i}=0$) as *fully independent* across all labs and replicates, that is, $\rho_{0|0}^{\left(l\right)}=0$. For genuine signals ($G_{i}=1$), we allow for *lab‐specific reproducibility*: if a signal is reproducible in lab l (i.e., $K_{i}^{\left(l\right)}=1$), then $\rho_{1|1}^{\left(l\right)}>0$; if it is irreproducible ($K_{i}^{\left(l\right)}=0$), then $\rho_{0|1}^{\left(l\right)}=0$. This allows the same signal to exhibit correlated structure in one lab but independent behavior in another, capturing the partial reproducibility patterns often seen in real multi‐source datasets. To ensure identifiability, we set $\mu_{0|0}^{\left(l\right)}=0$ and $\sigma_{0|0}^{\left(l\right)}=\sigma_{0|1}^{\left(l\right)}=1$. We assume $\mu_{1|1}^{\left(l\right)}>\mu_{0|1}^{\left(l\right)}>0$, reflecting that true and reproducible signals tend to yield stronger evidence. Lastly, note that the assumption of shared marginal distributions within a lab applies only to the latent variables z; the distributions of observed measurements x are allowed to vary across replicates.

Together, our model can be represented as a two‐step data generation process. First, the latent vector $z_{i}^{\left(l\right)}$ is generated from the nested Gaussian mixture model above. Then the observed data are generated by inverse transformation, $x_{i,m}^{\left(l\right)}=F_{m}^{(l)-1}(U^{\left(l\right)}(z_{i,m}^{\left(l\right)}))$, where $U^{\left(l\right)}$ is the marginal CDF of $z_{i,m}^{\left(l\right)}$ implied by the mixture, $U^{\left(l\right)}(z_{i,m}^{\left(l\right)})=\sum_{g=0}^{1}\pi_{g}\sum_{k=0}^{1}\pi_{k|g}^{\left(l\right)}\Phi ((z_{i,m}^{\left(l\right)}-\mu_{k|g}^{\left(l\right)})/\sigma_{k|g}^{\left(l\right)}).$

The full parameter set is: $\theta =[\pi_{1},{\left(\pi_{1|1}^{\left(l\right)},\mu_{0|1}^{\left(l\right)},\mu_{1|1}^{\left(l\right)},\sigma_{1|1}^{\left(l\right)},\rho_{1|1}^{\left(l\right)}\right)}_{l=1}^{L}].$ Substituting empirical CDFs for $F_{m}^{\left(l\right)}$, the likelihood is:

(2) $$\begin{array}{ll} L(\theta |X) & =\overset{n}{\underset{i=1}{\prod }}\overset{1}{\underset{g=0}{\sum }}\left(\pi_{g}\overset{L}{\underset{l=1}{\prod }}\overset{1}{\underset{k=0}{\sum }}\pi_{k|g}^{\left(l\right)}h_{k|g}^{\left(l\right)}(U^{(l)-1}(F_{1}^{\left(l\right)}(x_{i,1}^{\left(l\right)})),\right.\\ & \;\left.\ldots ,U^{(l)-1}(F_{M_{l}}^{\left(l\right)}(x_{i,M_{l}}^{\left(l\right)})))\right), \end{array}$$

where $h_{k|g}^{\left(l\right)}$ is the multivariate normal density defined above and $U^{(l)-1}$ is the inverse of the cumulative distribution induced by the mixture components. Parameter estimation is described in Section 2.2.

*Comparison to the original IDR model*: The original IDR model corresponds to the special case where L=1, treating all replicates as exchangeable. It does not distinguish between replicates from different sources and assumes a single‐level dependence structure. In contrast, nestedIDR explicitly models a two‐level structure: within‐lab reproducibility and across‐lab replicability. This distinction allows nestedIDR to more accurately capture the hierarchical and heterogeneous nature of real‐world multi‐source experiments, where lab‐specific variation and differing levels of reproducibility are common.

### 2.2 Measure of Reproducibility and Replicability

Given the parameter vector θ, we can determine the replicability and the reproducibility for each candidate. To assess the replicability for a candidate i across different data sources, we calculate the posterior probability that the candidate belongs to the noise group ($G_{i}=0$), which is defined as

$$\begin{array}{ll} idr_{i}^{\text{between}} & \equiv {\text{idr}}^{\text{between}}(x_{i}^{\left(l\right)},\ldots ,x_{i}^{\left(L\right)})\\ & =\frac{\begin{array}{l} \pi_{0}\prod_{l=1}^{L}\sum_{k=0}^{1}\pi_{k|0}^{\left(l\right)}h_{k|0}^{\left(l\right)}(U^{\left(l\right)}(F_{1}^{\left(l\right)}(x_{i,1}^{\left(l\right)})),\cdots \;,U^{\left(l\right)}(F_{M_{l}}^{\left(l\right)}(x_{i,M_{l}}^{\left(l\right)}))) \end{array}}{\begin{array}{l} \sum_{g=0}^{1}\left[\pi_{g}\prod_{l=1}^{L}\sum_{k=0}^{1}\pi_{k|g}^{\left(l\right)}h_{k|g}^{\left(l\right)}(U^{\left(l\right)}(F_{1}^{\left(l\right)}(x_{i,1}^{\left(l\right)})),\cdots \;,U^{\left(l\right)}(F_{M_{l}}^{\left(l\right)}(x_{i,M_{l}}^{\left(l\right)})))\right] \end{array}}. \end{array}$$

Using ${\text{idr}}^{\text{between}}$, one can quantify the replicability across sources for each candidate. Because candidates that are highly replicated across independent sources are likely to be real signals, this quantity can be thought of as a score that judges the authentication of the signals based on the replication information aggregated from all replicates across sources.

To assess the reproducibility of a replicable candidate across replicates from the same source, we calculate the posterior probability that the candidate is irreproducible ($K_{i}^{\left(l\right)}=0|G_{i}=1$) in source l given that it is replicable, which is defined as

$$\begin{array}{ll} & idr_{i}^{\text{within}(l)}\equiv {\text{idr}}^{\text{within}(l)}(x_{i}^{\left(l\right)})\\ & \;=\frac{\pi_{0|1}^{\left(l\right)}h_{0|1}^{\left(l\right)}({U^{\left(l\right)}}^{-1}(F_{1}^{\left(l\right)}(x_{i,1}^{\left(l\right)})),\ldots ,{U^{\left(l\right)}}^{-1}(F_{M_{l}}^{\left(l\right)}(x_{i,M_{l}}^{\left(l\right)})))}{\overset{1}{\underset{k=0}{\sum }}\pi_{k|1}^{\left(l\right)}h_{k|1}^{\left(l\right)}({U^{\left(l\right)}}^{-1}(F_{1}^{\left(l\right)}(x_{i,1}^{\left(l\right)})),\ldots ,{U^{\left(l\right)}}^{-1}(F_{M_{l}}^{\left(l\right)}(x_{i,M_{l}}^{\left(l\right)})))}. \end{array}$$

Using ${\text{idr}}^{\text{within}(l)}$, one can identify reproducible candidates from a given source and infer the overall data reproducibility for that source.

Furthermore, we define the expected rate of irreplicable discoveries for a set of candidates selected based on ${\text{idr}}^{\text{between}}$, analogous to that in the standard IDR framework [14], as

$$\begin{array}{l} {\text{IDR}}^{\text{between}}(\gamma )=\frac{\pi_{0}\int_{I_{\gamma }}dH_{0}(x_{i}^{\left(1\right)},\ldots ,x_{i}^{\left(L\right)})}{\int_{I_{\gamma }}dH(x_{i}^{\left(1\right)},\ldots ,x_{i}^{\left(L\right)})}, \end{array}$$

where $I_{\gamma }=\{(x_{i}^{\left(1\right)},\ldots ,x_{i}^{\left(L\right)}):{\text{idr}}^{\text{between}}(x_{i}^{\left(1\right)},\ldots ,x_{i}^{\left(L\right)})<\gamma \}$, $H_{g}$ and H are the CDF of the joint density functions

$$\begin{array}{ll} & h_{g}(x_{i}^{\left(1\right)},\ldots ,x_{i}^{\left(L\right)})\\ & \;=\overset{L}{\underset{l=1}{\prod }}\overset{1}{\underset{k=0}{\sum }}\pi_{k|g}^{\left(l\right)}h_{k|g}^{\left(l\right)}({U^{\left(l\right)}}^{-1}(F_{1}^{\left(l\right)}(x_{i,1}^{\left(l\right)})),\ldots ,{U^{\left(l\right)}}^{-1}(F_{M_{l}}^{\left(l\right)}(x_{i,M_{l}}^{\left(l\right)}))) \end{array}$$

and

$$h(x_{i}^{\left(1\right)},\ldots ,x_{i}^{\left(L\right)})=\overset{1}{\underset{g=0}{\sum }}\pi_{g}h_{g}(x_{i}^{\left(1\right)},\ldots ,x_{i}^{\left(L\right)}),$$

respectively. For a desired control level α, we denote the candidate ranked at the $i^{th}$ position by ${\text{idr}}^{\text{between}}$ values in an ascending order as $\left(x_{\left(i\right)}^{\left(1\right)},\ldots ,x_{\left(i\right)}^{\left(L\right)}\right)$ and define $s=\max\{i:\frac{1}{i}\sum_{j=1}^{i}idr_{j}^{\text{between}}\leq \alpha \}$. Then by selecting all $\left(x_{\left(i\right)}^{\left(1\right)},\ldots ,x_{\left(i\right)}^{\left(L\right)}\right)$ with i=1,...,s, this procedure gives an expected rate of irreplicable discoveries no greater than α.

Similarly, we define the expected rate of irreproducible discoveries in a set of candidates selected based on ${\text{idr}}^{\text{within}(l)}$ from the lab l as

$$\begin{array}{ll} & {\text{IDR}}^{\text{within}(l)}(\gamma )\;\\ & \;=\frac{\pi_{0|1}^{\left(l\right)}\int_{I_{\gamma }^{\left(l\right)}}dH_{0|1}^{\left(l\right)}({U^{\left(l\right)}}^{-1}(F_{1}^{\left(l\right)}(x_{i,1}^{\left(l\right)})),\ldots ,{U^{\left(l\right)}}^{-1}(F_{M_{l}}^{\left(l\right)}(x_{i,M_{l}}^{\left(l\right)})))}{\int_{I_{\gamma }^{\left(l\right)}}dH_{\cdot |1}^{\left(l\right)}({U^{\left(l\right)}}^{-1}(F_{1}^{\left(l\right)}(x_{i,1}^{\left(l\right)})),\ldots ,{U^{\left(l\right)}}^{-1}(F_{M_{l}}^{\left(l\right)}(x_{i,M_{l}}^{\left(l\right)})))},\; \end{array}$$

where $I_{\gamma }^{\left(l\right)}=\{x_{i}^{\left(l\right)}:{\text{idr}}^{\text{within}(l)}(x_{i}^{\left(l\right)})<\gamma \}$, $H_{k|1}^{\left(l\right)}$ and $H_{\cdot |1}^{\left(l\right)}$ are the CDF of density functions $h_{k|1}^{\left(l\right)}$ and $\sum_{k=0}^{1}\pi_{k|1}^{\left(l\right)}h_{k|1}^{\left(l\right)}$, respectively. For a desired control level α, we denote the candidate that is ranked at the ${\text{i}}^{\text{th}}$ position by ${\text{idr}}^{\text{within}(l)}$ values in an ascending order as $x_{\left(i\right)}^{\left(l\right)}$ and define $s^{\left(l\right)}=\max\{i:\frac{1}{i}\sum_{j=1}^{i}idr_{j}^{\text{within}(l)}\leq \alpha \}$. By selecting all $x_{\left(i\right)}^{\left(l\right)}$ with $i=1,...,s^{\left(l\right)}$, this procedure gives an expected rate of irreproducible discoveries no greater than α in lab l.

### 2.3 Estimation

We estimate the model parameters using a pseudo Expectation‐Maximization (EM) algorithm, adapted from the IDR approach [14]. Specifically, given an initial parameter set $\theta_{0}$, we first construct the pseudo data from the observed data using $U^{-1}\left(\frac{n}{n+1}{\hat{F}}_{m}^{\left(l\right)};\theta_{0}\right),$ where ${\hat{F}}_{m}^{\left(l\right)}$ is the empirical marginal CDF for replicate m in lab l, and the scaling factor n/(n+1) avoids boundary issues in the inverse normal transformation. We then alternate between the following two steps:

1. Parameter estimation based on pseudo‐data: Given the current pseudo‐data, we use the EM algorithm to maximize the complete‐data log‐likelihood (Equation 2) and update the parameter estimates θ.
2. Pseudo‐data update: Using the newly estimated parameters, we recompute the pseudo‐data as described above.

This iterative procedure is repeated until convergence. Convergence is declared when either (a) the relative change in likelihood is below a small threshold (as in [14]), or (b) the maximum change in parameter values across iterations falls below a predefined tolerance. Because the pseudo‐data transformation step may not increase the likelihood monotonically [23], we record the parameter estimates at each iteration and retain the one achieving the highest observed likelihood as the final estimate.

To improve robustness against local optima, we recommend running the algorithm from multiple initializations. Reasonable starting values can be chosen based on exploratory data analysis (e.g., empirical signal strengths or replicate correlations). Among these runs, the solution with the largest observed likelihood is selected. A detailed step‐by‐step description of the estimation procedure is provided in the Supporting Information, Section 1, Algorithm.

### 2.4 Computational Complexity and Runtime

NestedIDR's runtime is expected to scale approximately linearly with the number of labs, assuming a fixed number of replicates per lab. This follows from the structure of the likelihood function (Equation 2), which involves a product over labs and, within each lab, a sum over two reproducibility components. Each lab contributes a multivariate normal density term with its own set of parameters (e.g., $\mu_{k|g}^{\left(l\right)}$, $\sigma_{k|g}^{\left(l\right)}$, $\rho_{k|g}^{\left(l\right)}$), resulting in a total number of parameters that increases with the number of labs.

Model estimation is performed using an EM algorithm on pseudo‐data. In each E‐step, the likelihood is evaluated across all labs for every feature, leading to a runtime that scales approximately linearly with the number of features. Although the per‐feature computational cost grows with the number of labs, the total runtime is also influenced by convergence behavior and implementation details, such as vectorization and parallelization strategies.

## 3 Simulation Study

### 3.1 Baseline Simulations Under Model Assumptions

We first evaluated nestedIDR in a controlled setting where the data were generated exactly under the assumed nested copula mixture model. This baseline analysis isolates method performance from the confounding effects of model misspecification and directly assesses the benefits of modeling the nested structure. Specifically, we simulated data for two labs, each with two replicate samples, under varying conditions of signal‐to‐noise ratio and within‐lab variation. The signal‐to‐noise ratio was controlled by the proportion of noise $\pi_{0}$, and within‐lab variation was governed by $\sigma_{1|1}^{\left(2\right)}$ and $\rho_{1|1}^{\left(2\right)}$. We considered four settings (Table 1): (S1) high signal‐to‐noise ratio and low within‐lab variation for both labs; (S2) high signal‐to‐noise ratio with low within‐lab variation for one lab but high variation for the other; (S3) low signal‐to‐noise ratio and low within‐lab variation for both labs; and (S4) low signal‐to‐noise ratio with low variation for one lab but high variation for the other. S2 (S4) shares the same signal‐to‐noise ratio and lab 1 variation level as S1 (S3), but with higher variation in lab 2. This design allows us to disentangle the effects of global signal‐to‐noise ratio from those due to reproducibility issues in a single lab. For each setting, we generated 200 datasets, each with n=5000 candidates.

**TABLE 1:** True parameters for four simulation settings.

Scenario	$\pi_{0}$	$\pi_{0|1}^{\left(1\right)}$	$\pi_{0|1}^{\left(2\right)}$	$\mu_{0|1}^{\left(1\right)}$	$\mu_{0|1}^{\left(2\right)}$	$\mu_{1|1}^{\left(1\right)}$	$\mu_{1|1}^{\left(2\right)}$	$\sigma_{1|1}^{\left(1\right)}$	$\sigma_{1|1}^{\left(2\right)}$	$\rho_{1|1}^{\left(1\right)}$	$\rho_{1|1}^{\left(2\right)}$
S1	0.3	0.2	0.2	1.0	1.0	3.0	3.0	1.0	1.0	0.9	0.9
S2	0.3	0.2	0.5	1.0	1.0	3.0	3.0	1.0	1.4	0.9	0.7
S3	0.5	0.2	0.2	1.0	1.0	2.0	2.0	1.0	1.0	0.9	0.9
S4	0.5	0.2	0.5	1.0	1.0	2.0	2.0	1.0	1.4	0.9	0.7

We compared nestedIDR with:

- Standard IDR applied in two ways: (i) using all four replicates from both labs (${\text{idr}}^{a}$), which measures concordance across labs and serves as a replicability counterpart to ${\text{idr}}^{\text{between}}$ in nestedIDR, and (ii) using only replicates from a single lab (${\text{idr}}^{l_{1}}$ and ${\text{idr}}^{l_{2}}$), which measures within‐lab reproducibility and serves as a baseline for comparison.
- RankProd [18], a nonparametric meta‐analysis method for ranked lists, applied directly to the ranks of simulated data in each replicate.
- Meta *p*‐value methods, specifically Fisher's method [19] and Stouffer's method [20], which combine *p*‐values across replicates and labs; *p*‐values were obtained by transforming the simulated scores as described in the Supporting Information Section 2.

We also considered additional settings (S5–S6) with unequal numbers of replicates across labs and non‐exchangeable correlation structures within a lab, where one lab had three replicates with varying pairwise correlations and the other had two replicates (see Supporting Information Section 3.1 for details).

#### 3.1.1 Discrimination and Classification Accuracy Across Methods

Replicability reflects the likelihood that a signal is genuine. We evaluated each method's ability to (i) discriminate true signals from noise using the area under the ROC curve (AUC) and (ii) classify replicable signals at a fixed cutoff using the Adjusted Rand Index (ARI) [24]. For ARI computation, a candidate was classified as replicable if ${\text{idr}}^{\text{between}}\leq 0.05$ for nestedIDR, or if the corresponding IDR value ≤0.05 for the standard IDR‐based methods (${\text{idr}}^{a}$, ${\text{idr}}^{l_{1}}$, ${\text{idr}}^{l_{2}}$); otherwise it was classified as irreproducible. ARI measures agreement between the resulting classification and the truth, with a value of 1 for perfect agreement and an expected value of 0 under independence.

**FIGURE 3:**
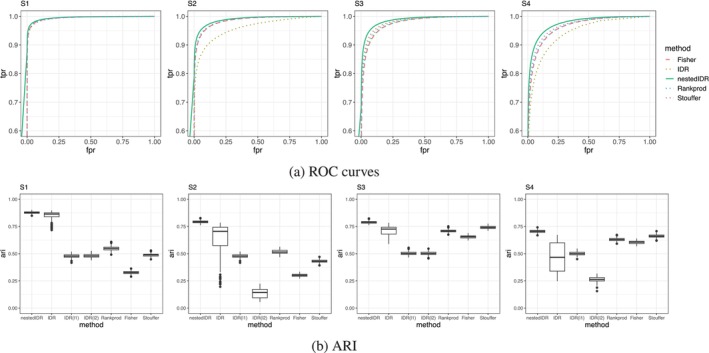
Comparison of five methods (nestedIDR, IDR, RankProd, Fisher, and Stouffer) in four simulation scenarios (S1 to S4). (a) ROC curves. Each ROC curve is the average over 200 simulation datasets. (b) Adjusted rand index (ARI) for classifying replicability of signals. IDR: IDR applied to all replicates; IDR(l1) and IDR(l2): IDR applied to replicates in lab 1 and lab 2, respectively.

Across all simulation settings (Table 2, Figure 3a), nestedIDR consistently achieved the highest AUC values. Multi‐lab methods (nestedIDR, ${\text{idr}}^{a}$, RankProd, Fisher's method, and Stouffer's method) generally outperformed single‐lab approaches (${\text{idr}}^{l_{1}}$, ${\text{idr}}^{l_{2}}$), whose accuracy depended heavily on the quality of the chosen lab. When labs had comparable quality and the signal‐to‐noise ratio was high (S1), all multi‐lab methods performed similarly. At lower signal‐to‐noise ratios (S3 and S4), nestedIDR's advantage became more pronounced, especially under heterogeneous lab quality. For example, in S2 and S4, nestedIDR markedly outperformed ${\text{idr}}^{a}$ (AUC: 0.989 vs. 0.957 in S2; 0.975 vs. 0.940 in S4), reflecting its ability to accommodate cross‐lab heterogeneity. While Fisher's and Stouffer's methods were less sensitive to heterogeneity than ${\text{idr}}^{a}$, they consistently lagged behind nestedIDR in accuracy.

**TABLE 2:** Comparison of AUC values under four simulation settings based on posterior probabilities from nestedIDR and IDR. ${\text{idr}}^{\text{between}}$: nestedIDR; ${\text{idr}}^{a}$: IDR applied to all four replicates jointly; ${\text{idr}}^{l_{1}}$ and ${\text{idr}}^{l_{2}}$: IDR applied separately to replicates from lab 1 and lab 2, respectively.

Scenario	nestedIDR	IDR			RankProd	Fisher	Stouffer
	${\text{idr}}^{\text{between}}$	${\text{idr}}^{a}$	${\text{idr}}^{l_{1}}$	${\text{idr}}^{l_{2}}$			
S1	0.995	0.993	0.937	0.938	0.994	0.994	0.994
S2	0.989	0.957	0.937	0.852	0.985	0.985	0.985
S3	0.986	0.978	0.912	0.919	0.976	0.975	0.982
S4	0.975	0.940	0.912	0.840	0.964	0.962	0.967

ARI results mirrored the AUC trends (Figure 3b). NestedIDR achieved the highest classification accuracy in all scenarios, with substantially lower variability across replicates than competing methods. Its advantage over ${\text{idr}}^{a}$ was most striking under heterogeneous lab quality, for example, S2 (SD of ARI: 0.012 for nestedIDR vs. 0.150 for ${\text{idr}}^{a}$) and S4 (0.012 vs. 0.136), underscoring the instability of ${\text{idr}}^{a}$ in such settings. RankProd, Fisher's method, and Stouffer's method also underperformed relative to nestedIDR in ARI, and the latter two were further hindered by the difficulty of selecting well‐calibrated *p*‐value cutoffs.

Parameter estimation results (Supporting Information Table 1) showed estimates consistently close to their true simulation values. Empirical runtimes for nestedIDR across S1–S4 and varying numbers of features are provided in Supporting Information Table 2. Results in simulations S5–S6 (Supporting Information Table 3; Supporting Information Figure 1) yielded patterns consistent with S1–S4, confirming nestedIDR's stability under varying replicate numbers and correlation structures. Collectively, these results demonstrate that explicitly modeling source‐specific variation yields superior discrimination and more stable classification in multi‐source studies.

#### 3.1.2 NestedIDR can Distinguish Between Different Causes of Aiscordance

Next we assessed the utility of our method as a tool for diagnosing data quality issues in multi‐lab studies. In particular, we evaluated whether our method could properly detect the reduction of concordance and illuminate the cause of said reduction when the concordance of findings across replicates is diminished. Here we focused on two common causes that can reduce the concordance of findings in multi‐lab studies: (1) low signal‐to‐noise ratios in the specimen distributed to all labs and (2) high variation in the measurement procedure of a lab. The former makes replication harder both within and across labs, thereby reducing both reproducibility and replicability. However, if the latter occurs in one lab, this has a larger impact on the reproducibility of the affected lab and a smaller impact on the overall replicability.

**FIGURE 4:**
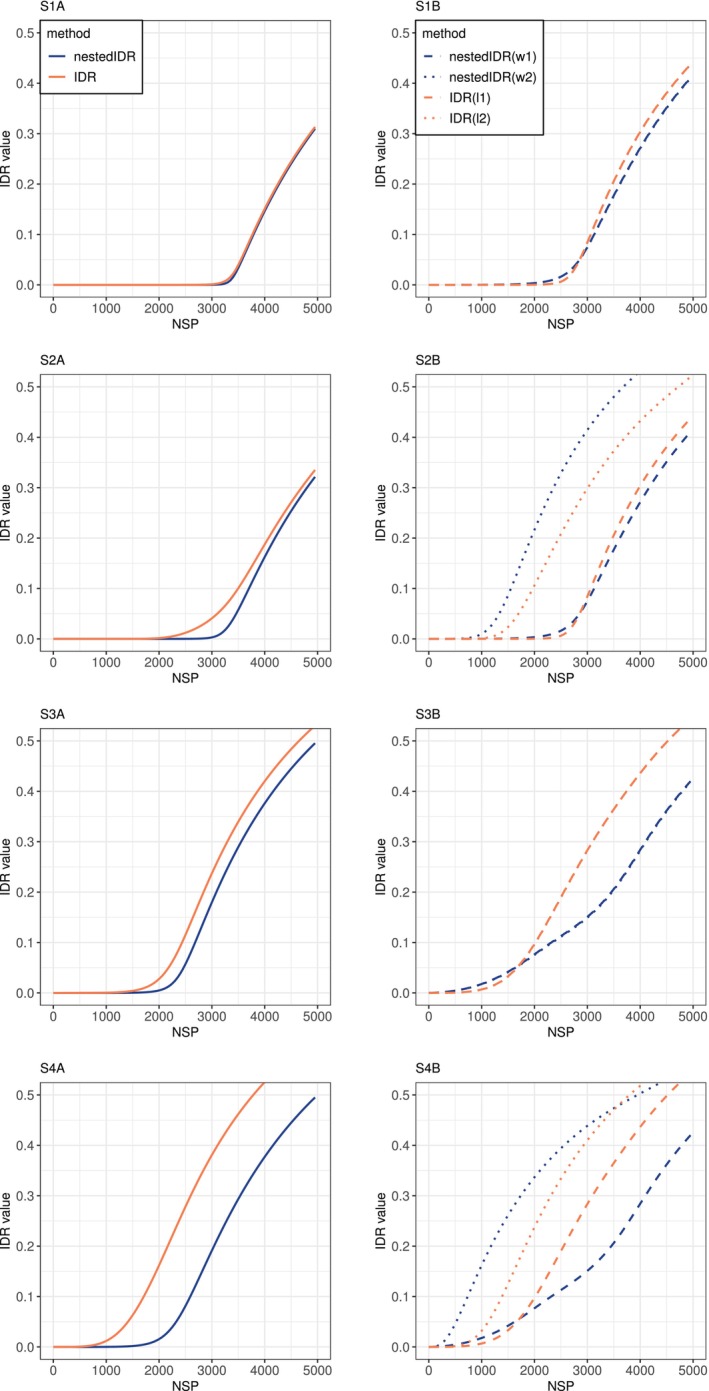
The number of selected signals at different reproducibility and replicability cutoffs in the four simulation settings (S1–S4). The left column shows the number of *replicable* signals selected using nestedIDR (${\text{idr}}^{\text{between}}$, blue solid) and IDR (${\text{idr}}^{a}$, orange solid) with data from both labs. The right column shows the number of *reproducible* signals selected using nestedIDR and IDR with the data from a single lab. nestedIDR(w1): lab 1 results from ${\text{idr}}^{\text{within}(1)}$ (blue dashed); nestedIDR(w2): lab 2 results from ${\text{idr}}^{\text{within}(2)}$ (blue dotted); IDR(l1): lab 1 results from ${\text{idr}}^{l_{1}}$ (orange dashed); and IDR(l2): lab 2 results from ${\text{idr}}^{l_{2}}$ (orange dotted).

To illustrate scenario (1), we consider the change from S1 to S3 and from S2 to S4. They represent the cases that the signal‐to‐noise ratio is reduced but the level of variation in the measurement procedure is intact for each lab, where the two labs have similar (or different, respectively) variation in S1 to S3 (S2 to S4, respectively). To illustrate scenario (2), we consider the change from S1 to S2 (or from S3 to S4, respectively). They represent the cases that the variation of lab 2 increases but the signal‐to‐noise ratio maintains the same high (or low, respectively) level. We compare the performance of nestedIDR and the standard IDR methods. To visualize the change of reproducibility or replicability at different cutoffs, we plotted the number of candidates selected by nestedIDR and the standard IDR method at a series of cutoffs (Figure 4). Given a replicability (or reproducibility) cutoff, a larger number of selected candidates indicates higher replicability (or reproducibility) of the data.

When the signal‐to‐noise ratio is lowered and the variation of measurement procedures remains intact, both nestedIDR (${\text{idr}}^{\text{between}}$, ${\text{idr}}^{\text{within}(l_{1})}$ and ${\text{idr}}^{\text{within}(l_{2})}$) and the standard IDR methods (${\text{idr}}^{a}$, ${\text{idr}}^{l_{1}}$ and ${\text{idr}}^{l_{2}}$) select substantially fewer signals with a reduction of 31.0‐41.8% at the cutoff of 0.05 (Figure 4 S1 vs. S3, or Figure 4 S2 vs. S4), correctly reflecting that the concordance is compromised beyond individual labs. When the signal‐to‐noise ratio is intact but the variation of measurement procedures from lab 2 increases (Figure 4 S1 vs. S2 and Figure 4 S3 vs. S4), the number of reproducible signals identified by ${\text{idr}}^{\text{within}(l_{2})}$ is substantially decreased (53.4% for S1 vs. S2 and 68.4% for S3 vs. S4), whereas the number of reproducible signals identified by ${\text{idr}}^{\text{within}(l_{1})}$ from lab 1 is unchanged and the numbers of replicable signals identified by ${\text{idr}}^{\text{between}}$ is only slightly reduced (3.14% and 5.68%, respectively).

This pattern signals that the lowered concordance is due to low reproducibility in lab 2, rather than the compromised signal‐to‐noise ratio. In contrast, while the standard IDR‐based method correctly reports lowered reproducibility in lab 2 (${\text{idr}}^{l_{2}}$), the number of replicable signals (identified by ${\text{idr}}^{a}$) is also greatly reduced (12.7% for S1 vs. S2 and 33.3% for S3 vs. S4), showing a pattern indifferent from the scenario of lowered signal‐to‐noise ratio. Together, this demonstrates the effectiveness of nestedIDR for differentiating the causes of reduced concordance and diagnosing data quality issues.

### 3.2 Robustness Simulations Under Assumption Violations

We next evaluated the robustness of nestedIDR when the data generation process deviated from the model assumptions, to better reflect complexities often encountered in real multi‐source experiments. Such deviations may arise from residual correlation between labs due to shared reagents or protocols, technical artifacts that induce dependence even among irreproducible measurements, or underlying signal structures that are not well captured by the assumed copula family.

We consider three scenarios: (1) cross‐lab dependence—violating the conditional independence assumption by introducing mild correlation between measurements from different labs; (2) within‐lab dependence for irreproducible signals—violating the assumption that irreproducible signals are independent within a lab by adding weak correlation among such replicates; (3) msspecified copula family—generating the reproducible components (k=1|g=1) from a Gumbel copula with dependence parameter θ=2 instead of the Gaussian copula assumed by nestedIDR. Unlike the Gaussian copula, which is symmetric with zero tail dependence, the Gumbel copula is asymmetric with zero lower‐tail dependence but positive upper‐tail dependence. This property allows it to represent data with particularly high correlation among top‐ranking signals.

In all scenarios, nestedIDR remained robust and consistently outperformed competing methods, including IDR and meta‐analysis approaches. The only notable difference under model misspecification was a slight shift in the ARI boxplots, with no substantial loss of performance.

## 4 Applications to Real Data

### 4.1 Application to the ENCODE ChIP‐seq Datasets

In our first analysis, we obtained ChIP‐seq data from the ENCODE project [1, 2]. According to ENCODE experimental design, for each binding protein, independent ChIP‐seq experiments were performed in two or more labs, with at least two biological replicates from each lab. Here we assessed the performance of nestedIDR using the ChIP‐seq experiments for the transcription factor CTCF on the K562 cell line. The data were generated by two labs, University of Texas at Austin (UTA) and University of Washington (UW), with two replicates contributed from each lab (UTA replicate 1: 4 861 426 reads, UTA replicate 2: 4 940 148 reads, UW replicate 1: 12 853 025 reads and UW replicate 2: 5 676 639 reads). We refer to UTA as lab 1 (l1) and UW as lab 2 (l2) in our analyses. We downloaded the BAM files directly from the ENCODE data portal [15, 21] and called ChIP‐seq peaks using MACS2 [25, 26] for each replicate sample. Due to variation across samples, the locations of ChIP‐seq peaks identified for the same binding event may be slightly different across replicates. We therefore merged the ChIP‐seq peaks that overlap across replicates using the bedtools [27] command ˜intercept˜. For each merged region, we used −log10(*p*‐value) calculated by MACS2 at the highest peaks within a merged region to represent the signal for the region. Then the reproducibility and replicability of the peaks across the replicates were evaluated using nestedIDR and the standard IDR methods based on these signals.

#### 4.1.1 NestedIDR Provides More Consistent Results With Motif Binding Scores

To validate the biological relevance of our results, we evaluated the motif occurrence at the identified ChIP‐seq peaks. A motif refers to a short DNA sequence that is preferentially bound by a binding protein. While true binding sites do not necessarily contain motifs, they generally have a higher frequency of motif occurrence than other regions. Here we obtained the FIMO score [28] for the binding sites identified in the ChIP‐seq experiments, which measures the likelihood of motif occurrence for each binding site, using the MEME suite [29]. Because signals in ChIP‐seq experiments reflect the experimentally measured likelihood of protein binding, the replicability of the ChIP‐seq peaks is expected to be positively correlated to their FIMO scores. We therefore evaluated the biological relevance of the replicability assessment by computing the Spearman correlation between the FIMO score and the replicability score assigned by each method. We observed that the results from nestedIDR have the higher Spearman correlation with the FIMO score (${\text{idr}}^{\text{between}}$: 0.440) than all the standard IDR methods (${\text{idr}}^{a}$: 0.425, ${\text{idr}}^{l1}$: 0.413 and ${\text{idr}}^{l2}$: 0.388). This demonstrates the effectiveness of nestedIDR in identifying biologically relevant signals.

#### 4.1.2 NestedIDR Enhances Support for Signals Replicated Across Labs Despite Within‐Lab Variability

An important benefit of multi‐source analyses is the ability to strengthen statistical support for findings by leveraging agreement across independent sources. For instance, when a signal appears variable or weak within one lab but is consistently observed in another independent lab, replication across labs can provide additional evidence in favor of the signal's authenticity.

To illustrate this, we compared the sets of ChIP‐seq peaks identified as replicable by nestedIDR versus the standard IDR model. Because both methods are based on mixture models, replicable signals can be defined as those with posterior probabilities below a threshold (${\text{idr}}^{\text{between}}$ for nestedIDR and ${\text{idr}}^{a}$ for standard IDR). We defined the nestedIDR‐specific set as peaks with ${\text{idr}}^{\text{between}}<0.3$ but ${\text{idr}}^{a}>0.6$, and the IDR‐specific set as peaks with ${\text{idr}}^{\text{between}}>0.6$ but ${\text{idr}}^{a}<0.3$, excluding ambiguous cases with intermediate scores (0.3−0.6) to focus on confident differences. This resulted in 710 peaks uniquely identified by nestedIDR and 773 uniquely identified by IDR.

**FIGURE 5:**
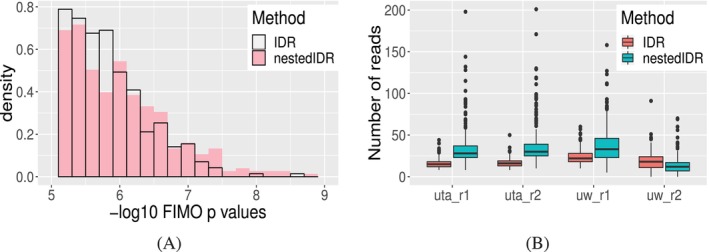
Comparison of the ChIP‐seq peaks that are specifically identified by nestedIDR (${\text{idr}}^{\text{between}}$) and IDR (${\text{idr}}^{a}$). (A) The distribution of FIMO −log10(*p*‐value) for peaks specifically identified by each method. (B) The distribution of read counts for the peaks specifically identified by each method in the four replicates.

**FIGURE 6:**
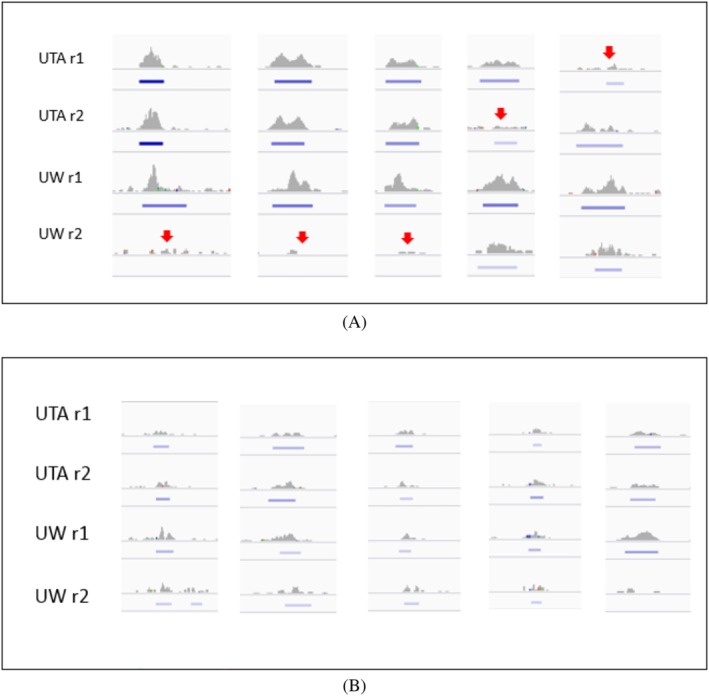
IGV Genome browser shots of example peaks (A) that are specifically identified by nestedIDR and (B) that are specifically identified by IDR (${\text{idr}}^{a}$). The ChIP‐seq data were obtained from two labs, UTA and UW. The Y axis shows the number of mapped reads, ranged 0‐40. The blue bar under each peak shows MACS2 peak calling results, where darker color indicates more significant MACS2 *p*‐values and no bar indicates that a peak is not called by MACS2. The red arrow marks the weakest peaks among the 4 replicates.

As shown in Figure 5A, both sets have similar FIMO score ranges, but the nestedIDR‐specific set contains a higher proportion of peaks with highly significant FIMO matches (e.g., 39.52% of nestedIDR‐specific peaks have $p<10^{-6}$, vs. 30.70% in the IDR‐specific set), suggesting stronger biological relevance. Further examination of the read count distributions (Figure 5B) revealed that IDR‐specific peaks tend to have moderate, consistent signals across all replicates. In contrast, nestedIDR‐specific peaks show strong signal in three replicates (UTA‐r1, UTA‐r2, and UW‐r1), but lower signal in one (UW‐r2). Visual inspection of representative peaks (Figure 6) confirms this pattern. As shown in Table 3, despite replication across multiple labs, these peaks are deemed irreplicable by the standard IDR model (${\text{idr}}^{a}$), which assumes homogeneous replicates and thus penalizes inconsistent signals. In contrast, nestedIDR, which models lab‐specific variability, assigns high replicability scores (${\text{idr}}^{\text{between}}$) to these peaks.

**TABLE 3:** Comparison of ChIP‐seq peaks uniquely identified by nestedIDR and IDR for the example regions shown in Figure 6.

Peak No.	Chr	Start	End	FIMO	nestedIDR	IDR			
				*p*	${\text{idr}}^{\text{between}}$		${\text{idr}}^{a}$	${\text{idr}}^{l_{1}}$	${\text{idr}}^{l_{2}}$
Peaks specifically selected by ${\text{idr}}^{\text{between}}$									
1	1	10 927 230	10 927 723	0.0e−05	0.00		0.99	0.00	0.99
2	1	667 190 74	66 719 292	5.5e−05	0.00		0.99	0.00	0.99
3	1	95 186 469	95 186 656	0.7e−05	0.00		0.99	0.00	0.98
4	1	156 163 732	156 163 945	0.1e−05	0.07		0.68	1.00	0.06
5	15	74 220 059	74 220 482	2.7e−05	0.28		0.61	0.99	0.01
Peaks specifically selected by ${\text{idr}}^{a}$									
1	1	9 633 864	9 634 107	0.2e−05	0.63		0.01	0.79	0.90
2	1	37 078 559	37 078 824	0.3e−05	0.83		0.03	0.99	0.99
3	3	133 629 334	133 629 568	6.0e−05	0.92		0.09	0.99	0.95
4	7	104 585 490	104 585 725	5.7e−05	0.60		0.01	0.96	0.90
5	14	24 092 316	24 092 504	1.3e−05	0.60		0.01	0.96	0.90

*Note:* For each peak, we report its genomic location, FIMO motif p‐value, and posterior probabilities of being a genuine signal under different models: ${\text{idr}}^{\text{between}}$ from nestedIDR; ${\text{idr}}^{a}$ from IDR applied to all four replicates jointly; ${\text{idr}}^{l_{1}}$ and ${\text{idr}}^{l_{2}}$ from IDR applied separately to the two replicates from UTA and UW, respectively.

These observations highlight how nestedIDR increases support for signals that are replicated across labs but suppressed by within‐lab variability. The contrasting behaviors of the two methods stem from their modeling assumptions: standard IDR treats all replicates as exchangeable and favors consistency across all replicates. While this is effective for homogeneous datasets, it can lead to false negatives in multi‐source contexts where technical variation differs between labs. In contrast, nestedIDR accommodates source‐specific heterogeneity, allowing it to distinguish signals that are irreproducible due to lab‐specific noise from those that are not replicated across sources.

### 4.2 Application on the SEQC RNA‐seq Data

**FIGURE 7:**
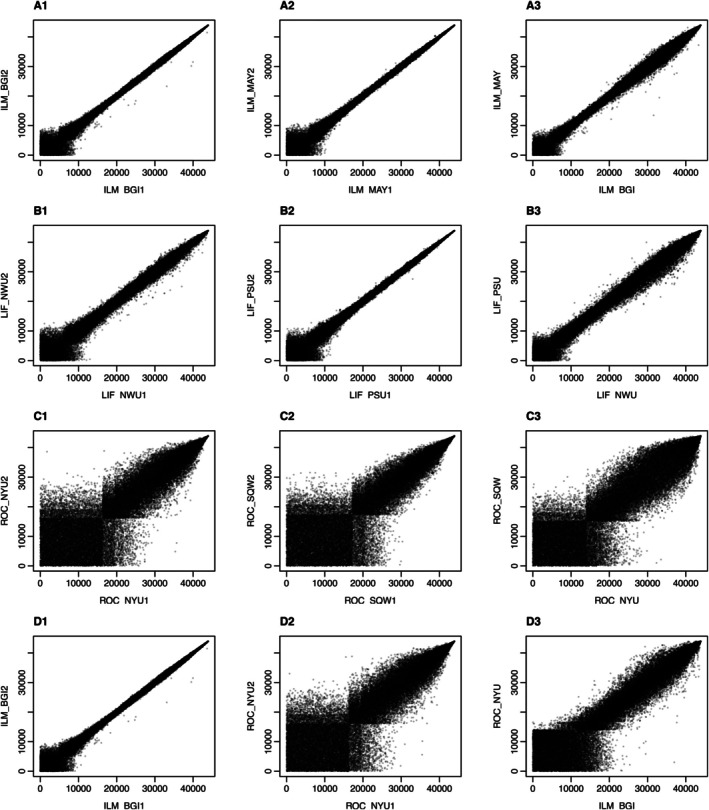
SEQC RNA‐seq data. Plotted are the rank scatterplots of the gene expression level (read counts) measured using different technologies (Illumina (ILM), Life technologies (LIF) and Roche (ROC)) in different labs. Rows A‐C: within‐platform analysis; Row D: across‐platforms analysis. Specifically, A: ILM (BGI) vs. ILM (MAY); B: LIF (NWU) vs. LIF (PSU); C: ROC (NYU) vs. ROC (SQW); D: ILM (BGI) vs. ROC (NYU). Columns 1 and 2: two replicates from each participating labs. Column 3: the average expression level of the two labs in the first two columns. The average expression level is calculated as the average between the two replicates in each lab.

Next we applied nestedIDR to the RNA‐seq data from the Sequencing Quality Control (SEQC) project [22]. SEQC compared the performance of three RNA‐seq platforms using benchmark datasets for Universal Human Reference RNA sample and Human Brain RNA sample (see Figure 7). Each RNA sample was sequenced using three platforms, namely, Illumina HiSeq 2000, Life Technologies and Roche 454. Illumina HiSeq 2000 was used to generate data at the Mayo Clinic (MAY), BGI, Cornell, City of Hope, Novartis and the Australian Genome Research Facility. Life Technologies was used to generate data at Penn State (PSU), Northwestern (NWU), SeqWright Inc. (SQW), and Liverpool. Lastly, Roche 454 was used to generate data at the Medical Genomes Project, New York University Medical Center (NYU), and SeqWright Inc. The consortium also measured the gene expression levels for a subset of 1129 genes using the qRT‐PCR experiment, which often is deemed as a gold standard to evaluate the accuracy of RNA measurements.

Here we assessed the replicability and reproducibility of the gene expression levels using the Universal Human Reference RNA sample. Our analyses focus on evaluating the between‐lab replicability and within‐lab reproducibility for each platform. In particular, for each platform, we selected two labs (Table 4 rows 1–3), and within each lab, we used two replicate samples for data from each platform. Figure 7A–C shows the rank scatter plots of the read counts between replicate samples from the same lab (Figure 7A1‐2, B1‐2, C1‐2) and from different labs (Figure 7A3, B3, C3). As shown, Illumnia and Life technologies consistently show much higher reproducibility between replicate samples than Roche. We applied nestedIDR and the standard IDR to the read counts generated from each RNA‐seq platform. In addition, to evaluate the robustness of our method in presence of high heterogeneity, we also performed an across‐platform analysis (Figure 7D) by comparing data generated from Illumina HiSeq 2000 at BGI with that from Roche 454 at NYU (Table 4 row 4). Similar to the within‐platform analysis, we used two replicate samples within each lab. Because both lab and platform are different between the two replicate samples in the across‐platform analysis, the replicates are more heterogeneous than those in the within‐platform analysis.

**TABLE 4:** Spearman rank correlation between replicability metrics and the PCR measurements.

Platform	Labs	nestedIDR	IDR		
		${\text{idr}}^{\text{between}}$	${\text{idr}}^{a}$	${\text{idr}}^{l_{1}}$	${\text{idr}}^{l_{2}}$
Illumina	BGI	0.8593	0.8189	0.8164	0.8148
Illumina	MAY				
Life technologies	PSU	0.8903	0.8470	0.8768	0.8801
Life technologies	NWU				
Roche 454	NYU	0.8717	0.8378	0.8571	0.8221
Roche 454	SQW				
Illumina	BGI	0.8854	0.8006	0.8164	0.8571
Roche 454	NYU				

*Note:* Metrics include posterior probabilities from nestedIDR (${\text{idr}}^{\text{between}}$), IDR on all four replicates jointly (${\text{idr}}^{a}$), and IDR applied separately to the first (${\text{idr}}^{l_{1}}$) and second (${\text{idr}}^{l_{2}}$) platform‐lab combinations.

#### 4.2.1 NestedIDR Shows a High Correlation With Expression Levels Measured by PCR

To evaluate the biological relevance of our assessments, we compared the results from each method with the PCR measured expression value. Although PCR measurements are only available for a small subset of the genes considered in the RNA‐seq datasets (1129 out of more than 40 000 transcripts), they nevertheless provide some insights. Because an authentic signal is expected to be more replicable and stronger than noise, the ranking provided by a good replicability metric is expected to be reasonably concordant with the ranking of the true signal strength. Based on this intuition, we computed the Spearman rank correlation between the estimated metrics and PCR measurements.

As shown in Table 4, nestedIDR has the highest Spearman rank correlation with the PCR measurements in all cases. We observed that ${\text{idr}}^{a}$ sometimes performs worse than both single‐lab results (${\text{idr}}^{l1}$ and ${\text{idr}}^{l2}$) (Table 4 rows 2 and 4), especially when different technologies are used in the two labs (Table 4 row 4). This is likely because the homogeneous assumption in the standard IDR method (${\text{idr}}^{a}$) is violated when there is a high level of between‐lab heterogeneity. In this situation, the integration across labs using the standard IDR can lead to results that are even worse than those obtained by only using the data from a single lab. In contrast, nestedIDR consistently outperforms the single‐lab results in all cases, even when the data come from different platforms (Table 4 row 4), showing the robustness and effectiveness of nestedIDR in integrating data from heterogeneous sources.

## 5 Conclusions

In this paper, we developed nestedIDR, a nested copula mixture model for assessing the reproducibility and replicability of high‐throughput genomic data across multiple sources. By modeling the dependence between replicate samples according to their source‐specific origins, nestedIDR captures the structured variability introduced by nested experimental designs commonly seen in consortium efforts or independently replicated studies. This framework effectively accounts for between‐source heterogeneity while simultaneously estimating both within‐source reproducibility and between‐source replicability.

Note that nestedIDR is different from applying standard IDR to batch‐corrected data. NestedIDR explicitly captures the hierarchical dependence of within‐from between‐source variation through lab‐specific parameters, enabling separate estimation of reproducibility and replicability. The latter still treats all replicates as exchangeable and thus cannot recover the same source‐specific insights as nestedIDR.

Importantly, nestedIDR is not equivalent to applying standard IDR to batch‐corrected data. While batch‐correction methods aim to remove systematic shifts, they do not model the hierarchical dependence structure of nested designs. NestedIDR explicitly incorporates lab‐specific parameters to distinguish within‐lab from between‐lab variation, enabling separate estimation of reproducibility and replicability‐insights that standard IDR, even after batch correction, cannot provide.

Our analyses of ChIP‐seq and RNA‐seq datasets demonstrate that the replicability scores from nestedIDR align more closely with true signal strength than those from standard IDR. In particular, nestedIDR better tolerates signals that may be weakly reproducible within individual labs but are consistently observed across labs, thereby recovering biologically meaningful signals that would otherwise be missed. The method is broadly applicable, not only to genomic data but also to other high‐throughput experiments such as fMRI studies identifying brain activity patterns.

## Funding

This work was supported by the National Institutes of Health (Grant Nos. R01GM109453 to Q.L. and F31HG010574 to H.K.).

## Conflicts of Interest

The authors declare no conflicts of interest.

## Supporting information




**Data S1.** Supporting Information.

## Data Availability

The data that support the findings of this study are available in ENCODE at https://www.encodeproject.org/. These data were derived from the following resources available in the public domain: ENCODE, https://www.encodeproject.org/SEQC, https://www.ncbi.nlm.nih.gov/gds/?term=GSE47792. The nestedIDR R package, including simulation scripts, datasets for reproducing the real‐data analyses, and full documentation, is publicly available on GitHub (https://github.com/qunhualilab/nestedIDR).
