# Supplementary material for: A Statistical Framework for Measuring Reproducibility and Replicability of High‐Throughput Experiments From Multiple Sources
Source: Stat Med. 2026 Feb 4;45(3-5):e70354. doi: 10.1002/sim.70354 (PMC12871010; doi:10.1002/sim.70354)
Supplement: Supplementary file 1 — Data S1. Supporting Information. [file SIM-45-0-s001.pdf]

Supplementary Material to: A statistical framework for measuring reproducibility and replicability of high-throughput experiments from multiple sources

Monia Ranalli†, Yafei Lyu†, Hillary Koch, Qunhua Li

1 Algorithm

1. Perform a preliminary exploratory analysis to determine the possible range of values for each parameter (θ_0).
2. Run the following algorithm from multiple starting values, and select the estimates that achieve the maximum observed likelihood value as the final estimates.
3. Compute the pseudo-data $U^{(l)-1}\left(\frac{n}{n+1}\hat{F}_m^{(l)};\theta_0\right)$ from some initialization parameters θ_0, where $\hat{F}_m^{(l)}$ is the empirical marginal CDF and $n/(n+1)$ is a rescaling factor to avoid infinities.
4. **Estimation.** The mixture likelihood for the data is given by:

$$L(\theta)=\prod_{i=1}^{n}\sum_{g=0}^{1}\left(\pi_g\prod_{l=1}^{L}\sum_{k=0}^{1}\pi_{k|g}^{(l)}h_{k|g}^{(l)}\left(U^{(l)-1}\left(F_1^{(l)}(x_{i,1}^{(l)}),\ldots,F_M^{(l)}(x_{i,M}^{(l)})\right)\right)\right)$$

where $h_{k|g}^{(l)}$ is the multivariate normal density with parameters $\mu_{k|g}^{(l)}$, $\sigma_{k|g}^{(l)}$, and $\rho_{k|g}^{(l)}$.

The parameter vector θ is estimated using a pseudo-likelihood method, assuming:

$$\mu_{0|0}^{(l)}=0,\quad \mu_{1|1}^{(l)}>\mu_{0|1}^{(l)}>0,\quad \rho_{0|0}^{(l)}=\rho_{0|1}^{(l)}=0,\quad \rho_{1|1}^{(l)}>0,\quad \sigma_{0|0}^{(l)}=\sigma_{0|1}^{(l)}=1$$

The complete log-likelihood:

$$\begin{aligned}\ell_c(\theta)=&\sum_{i=1}^{n}\sum_{g=0}^{1}w_{ig}\log\pi_g+\sum_{i=1}^{n}\sum_{g=0}^{1}\sum_{l=1}^{L}\sum_{k=0}^{1}w_{ig}w_{ik|g}^{(l)}\log\pi_{k|g}^{(l)}\\&+\sum_{i=1}^{n}\sum_{g=0}^{1}\sum_{l=1}^{L}\sum_{k=0}^{1}w_{ig}w_{ik|g}^{(l)}\log h_{k|g}^{(l)}\left(U^{(l)-1}\left(F_1^{(l)}(x_{i,1}^{(l)}),\ldots,F_M^{(l)}(x_{i,M}^{(l)})\right)\right)\end{aligned}$$

The EM algorithm:

- **E-step:**

$$w_{ig}=\frac{\pi_g\prod_{l=1}^{L}\sum_{k=0}^{1}\pi_{k|g}^{(l)}h_{k|g}^{(l)}\left(U^{(l)-1}\left(F_1^{(l)}(x_{i,1}^{(l)}),\ldots,F_M^{(l)}(x_{i,M}^{(l)})\right)\right)}{\sum_{g=0}^{1}\pi_g\prod_{l=1}^{L}\sum_{k=0}^{1}\pi_{k|g}^{(l)}h_{k|g}^{(l)}\left(U^{(l)-1}\left(F_1^{(l)}(x_{i,1}^{(l)}),\ldots,F_M^{(l)}(x_{i,M}^{(l)})\right)\right)}$$

[0]†Co-first authors.

$$w_{ik|g}^{(l)} = \frac{\pi_{k|g}^{(l)} h_{k|g}^{(l)} \left(U^{(l)-1}\left(F_1^{(l)}(x_{i,1}^{(l)}), \ldots, F_M^{(l)}(x_{i,M}^{(l)})\right)\right)}{\sum_{k=0}^{1} \pi_{k|g}^{(l)} h_{k|g}^{(l)} \left(U^{(l)-1}\left(F_1^{(l)}(x_{i,1}^{(l)}), \ldots, F_M^{(l)}(x_{i,M}^{(l)})\right)\right)}$$

- **M-step:**

$$\hat{\pi}_g = \frac{\sum_{i=1}^{n} w_{ig}}{n}$$

$$\hat{\pi}_{k|g}^{(l)} = \frac{\sum_{i=1}^{n} w_{ik|g}^{(l)} w_{ig}}{n \hat{\pi}_g}$$

$$\hat{\mu}_{k|g}^{(l)} = \frac{\sum_{i=1}^{n} w_{ig} w_{ik|g}^{(l)} \sum_{m=1}^{M} z_{i,m}^{(l)}}{M \sum_{i=1}^{n} w_{ig} w_{ik|g}^{(l)}}$$

$$\hat{\sigma}_{k|g}^{(l)} = \sqrt{\frac{\sum_{i=1}^{n} w_{ig} w_{ik|g}^{(l)} \sum_{m=1}^{M} \left(z_{i,m}^{(l)} - \hat{\mu}_{k|g}^{(l)}\right)^2}{M \sum_{i=1}^{n} w_{ig} w_{ik|g}^{(l)}}}$$

$$\hat{\rho}_{k|g}^{(l)} = \frac{2 \sum_{i=1}^{n} w_{ig} w_{ik|g}^{(l)} \sum_{m=1}^{M-1} \sum_{m'=2}^{M} \left(z_{i,m}^{(l)} - \hat{\mu}_{k|g}^{(l)}\right) \left(z_{i,m'}^{(l)} - \hat{\mu}_{k|g}^{(l)}\right)}{\sum_{i=1}^{n} w_{ig} w_{ik|g}^{(l)} \left(\sum_{m=1}^{M-1} \left(z_{i,m}^{(l)} - \hat{\mu}_{k|g}^{(l)}\right)^2 + \sum_{m'=2}^{M} \left(z_{i,m'}^{(l)} - \hat{\mu}_{k|g}^{(l)}\right)^2\right)}$$

5. Compute the log-likelihood of the copula mixture model in step (4).
6. Update the pseudo-data based on the parameter estimates from step (3).
7. Repeat E-step and M-step until convergence (e.g., change in log-likelihood $< \varepsilon$ or parameter change $< \varepsilon$).

2 Supplementary Notes for Section 3.1 Baseline simulation under model assumptions

2.1 P-value Generation for Meta-analysis Methods

For Fisher's and Stouffer's methods, which require p-values as input, we derived p-values from the simulated data as follows. For each simulated latent variable z, we obtained a measurement value x via $x = t_5^{-1}(U(z))$, where $t_5(\cdot)$ is the CDF of a t-distribution with 5 degrees of freedom and U is the marginal CDF of z implied by the mixture. We then performed a one-sided z-test for the alternative hypothesis $H_1 : x > 0$ to obtain a p-value for each replicate. For each candidate, these replicate-level p-values were combined across all replicates and labs using Fisher's method, which sums the log-transformed p-values, $T_F = -2\sum_{j=1}^{m} \log(p_j)$, and Stouffer's method, which aggregates z-scores under the assumption of independence $T_S = \frac{\sum_{j=1}^{m} \Phi^{-1}(1-p_j)}{\sqrt{m}}$, where m is the total number of p-values combined and $\Phi^{-1}(\cdot)$ is the quantile function of the standard normal distribution. These approaches treat replicates as independent and do not account for the nested correlation structure in the data.

2.2 Parameter estimation and runtime for simulations S1-S4

The parameters estimated using nestedIDR for S1-S4 are summarized in Supplementary Table 1 for 200 simulations at $N = 5000$. The runtime of nestedIDR is summarized in Supplementary Table 2.

Supplementary Table 1: Means, standard deviations and MSEs across the four scenarios (S1-S4).

	S1			S2			S3			S4		
	MSE	Mean	SD	MSE	Mean	SD	MSE	Mean	SD	MSE	Mean	SD
π_1	0.0001	0.6998	0.0073	0.0001	0.7003	0.0073	0.0001	0.4996	0.0080	0.0001	0.5000	0.0092
$\pi_{0\|1}^{(1)}$	0.0001	0.7982	0.0119	0.0002	0.7979	0.0130	0.0003	0.7974	0.0170	0.0003	0.7967	0.0180
$\pi_{0\|1}^{(2)}$	0.0001	0.7990	0.0115	0.0002	0.4998	0.0135	0.0002	0.7992	0.0157	0.0005	0.4994	0.0223
$\mu_{0\|1}^{(1)}$	0.0011	1.0024	0.0331	0.0012	1.0017	0.0349	0.0014	0.9984	0.0373	0.0017	0.9997	0.0414
$\mu_{0\|1}^{(2)}$	0.0012	1.0030	0.0344	0.0008	1.0012	0.0276	0.0012	0.9988	0.0353	0.0011	0.9996	0.0329
$\mu_{1\|1}^{(1)}$	0.0005	3.0015	0.0215	0.0005	3.0011	0.0229	0.0006	2.0008	0.0248	0.0007	2.0015	0.0258
$\mu_{1\|1}^{(2)}$	0.0004	3.0001	0.0200	0.0003	2.9976	0.0175	0.0006	2.0006	0.0242	0.0007	1.9985	0.0261
$\sigma_{1\|1}^{(1)}$	0.0005	0.9890	0.0203	0.1687	0.9897	0.0190	0.0006	0.9905	0.0228	0.1674	0.9913	0.0202
$\sigma_{1\|1}^{(2)}$	0.0006	0.9897	0.0216	0.0003	1.3910	0.0160	0.0006	0.9903	0.0233	0.0012	1.3841	0.0313
$\rho_{1\|1}^{(1)}$	0.0000	0.8994	0.0039	0.0000	0.8994	0.0040	0.0000	0.8992	0.0049	0.0000	0.8997	0.0053
$\rho_{1\|1}^{(2)}$	0.0000	0.8997	0.0038	0.0002	0.7022	0.0138	0.0000	0.8995	0.0051	0.0004	0.7019	0.0206

Supplementary Table 2: Elapsed time (in seconds) for simulations S1-S4 at different values of N. Processor: Intel(R) Core(TM) i7-10750H CPU @ 2.60GHz, 2592 Mhz, 6 Core(s), 12 Logical Processor(s)

**Scenario**	**N = 5000**	**N = 10000**	**N = 15000**	**N = 20000**
S1	16.07	15.12	21.71	36.34
S2	11.85	13.89	21.92	34.94
S3	16.67	29.37	36.26	66.49
S4	19.30	30.23	41.33	58.43

3 Additional simulations

3.1 Simulations with unequal numbers of replicates across labs and non-exchangeable correlation structures within labs

To assess the flexibility of nestedIDR under more complex experimental designs, we considered simulations (S5–S6) with unequal numbers of replicates across labs and non-exchangeable correlation structures within a lab. In both settings, lab 1 had three replicates and lab 2 had two replicates. In S5, the signal-to-noise ratio matched that of S1, and the three replicates in lab 1 had varying pairwise correlations $\rho_{1|1}^{(12;1)}$, $\rho_{1|1}^{(23;1)}$, and $\rho_{1|1}^{(13;1)}$. In S6, the signal-to-noise ratio matched that of S3, and the three replicates in lab 1 had equal pairwise correlations (exchangeable $\rho_{1|1}^{(2)}$). This design evaluates nestedIDR's performance when both the number of replicates and the correlation structures differ across labs.

As shown in Supplementary Table 3, the estimated parameters remain close to their true values. Supplementary Figure 1 further demonstrates that nestedIDR maintains strong performance under these conditions, consistently outperforming IDR and meta-analysis methods (RankProd, Fisher's method, and Stouffer's method) in both true positive rate and classification accuracy. These findings underscore the practical utility of nestedIDR for real-world studies where replicate numbers are unbalanced or correlation structures are heterogeneous.

Supplementary Table 3: True parameters and estimated means, standard deviations, and MSEs for two simulation settings (S5 and S6). Both settings have two labs, where lab 1 has three replicates and lab 2 has two replicates.

	S5				S6			
	True parameter	MSE	Mean	SD	True parameter	MSE	Mean	SD
π_1	0.7	0.0000	0.7001	0.0063	0.5	0.0001	0.5002	0.0083
$\pi_{1\|1}^{(1)}$	0.8	0.0001	0.8010	0.0095	0.8	0.0001	0.7997	0.0114
$\pi_{1\|1}^{(2)}$	0.8	0.0005	0.7806	0.0115	0.8	0.0005	0.7843	0.0161
$\mu_{1\|1}^{(1)}$	3.0	0.0024	2.9731	0.0413	2.0	0.0035	1.9931	0.0588
$\mu_{1\|1}^{(2)}$	3.0	0.0016	3.0347	0.0203	2.0	0.0015	2.0131	0.0359
$\mu_{0\|1}^{(1)}$	1.0	0.0013	0.9909	0.0356	1.0	0.002	1.0067	0.0446
$\mu_{0\|1}^{(2)}$	1.0	0.0021	1.0256	0.0381	1.0	0.0088	0.919	0.0471
$\sigma_{1\|1}^{(1)}$	1.0	0.0008	0.9924	0.0266	1.0	0.0012	0.995	0.0338
$\sigma_{1\|1}^{(2)}$	1.0	0.0100	0.9003	0.0044	1.0	0.0100	0.9002	0.0045
$\rho_{1\|1}^{(12;1)}$	0.9	0.0000	0.9003	0.0041	0.9	0.0000	0.8994	0.0043
$\rho_{1\|1}^{(13;1)}$	0.9	0.0001	0.8934	0.0043	0.9	0.0001	0.8929	0.0052
$\rho_{1\|1}^{(23;1)}$	0.7	0.0001	0.7014	0.117	0.9	0.0002	0.899	0.0137
$\rho_{1\|1}^{(12;2)}$	0.9	0.0044	0.9651	0.0325	0.9	0.0001	0.9081	0.0071

3.2 Simulations for testing the robustness of NestedIDR to violations of within-lab independence for irreproducible genuine signals

The nestedIDR model assumes that irreproducible signals within a lab are independent across replicates. While this assumption is reasonable in many settings, slight dependence may arise due to

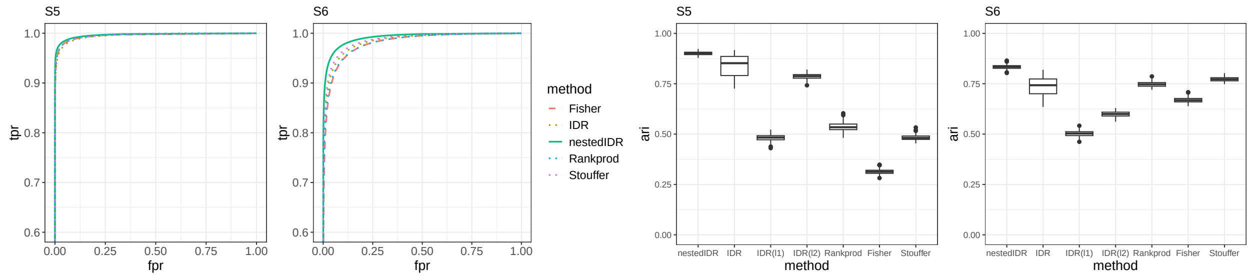

Supplementary Figure 1: Comparison of nestedIDR, IDR, RankProd, Fisher, and Stouffer in simulations S5–S6. These simulations involve two labs—one with three replicates exhibiting varying pairwise correlations and the other with two replicates. Left two columns: ROC curves; right two columns: ARI values.

shared experimental conditions or systematic noise.

To assess the robustness of nestedIDR under violations of this assumption, we conducted a set of simulations (denoted S1–S4) in which irreproducible genuine signals ($K = 0 \mid G = 1$) were weakly correlated within each lab. Specifically, we introduced a within-lab correlation of $\rho_{0|1} = 0.2$ between the two replicates in each of two labs. This correlated structure was applied to each of the four simulation scenarios (S1–S4) described in the main text.

The simulation parameters are summarized in Supplementary Table 4. Results shown in Supplementary Figure 2a demonstrate that nestedIDR maintains the highest true positive rates across all scenarios, outperforming IDR, RankProd, Fisher's method, and Stouffer's method. While the performance of IDR and meta-analysis methods degrades under this form of model misspecification, nestedIDR remains robust and continues to yield reliable signal detection.

Supplementary Table 4: True parameters used in simulations that violate the model assumption of within-lab independence for irreproducible genuine signals.

Scenario	π_1	$\pi_{0\|1}^{(1)}$	$\pi_{0\|1}^{(2)}$	$\mu_{0\|1}^{(1)}$	$\mu_{0\|1}^{(2)}$	$\mu_{1\|1}^{(1)}$	$\mu_{1\|1}^{(2)}$	$\sigma_{1\|1}^{(1)}$	$\sigma_{1\|1}^{(2)}$	$\rho_{0\|1}^{(1)}$	$\rho_{0\|1}^{(2)}$	$\rho_{1\|1}^{(1)}$	$\rho_{1\|1}^{(2)}$
S1*	0.7	0.2	0.2	1	1	3	3	1	1.0	0.2	0.2	0.9	0.9
S2*	0.7	0.2	0.5	1	1	3	3	1	1.4	0.2	0.2	0.9	0.7
S3*	0.5	0.2	0.2	1	1	2	2	1	1.0	0.2	0.2	0.9	0.9
S4*	0.5	0.2	0.5	1	1	2	2	1	1.4	0.2	0.2	0.9	0.7

3.3 Simulations for evaluating the robustness of NestedIDR to violations of cross-lab independence

The nestedIDR model assumes conditional independence of replicates across labs given the latent signal status. This assumption reflects the typical structure of multi-lab studies, where experiments are conducted independently using different personnel, instruments, or protocols.

To evaluate the robustness of nestedIDR to violations of this assumption, we conducted an additional simulation study in which replicates from different labs exhibit mild correlation. Specifically, we introduced cross-lab dependence by modifying the measurements as

$$x_i^{(2)} = x_i^{(2)} + 0.2x_i^{(1)},$$

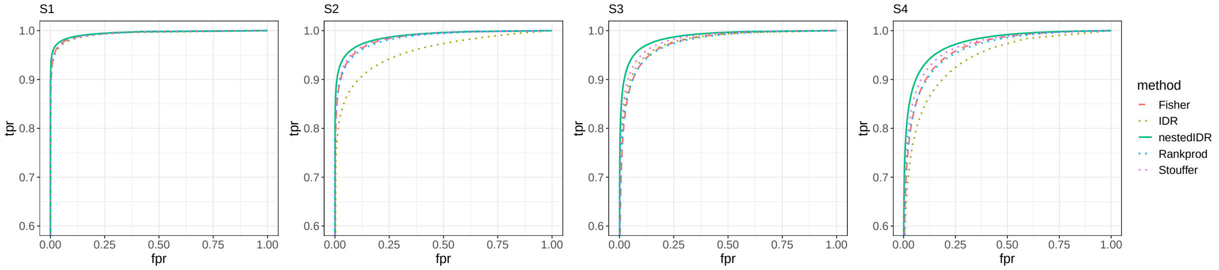

(a) ROC curve

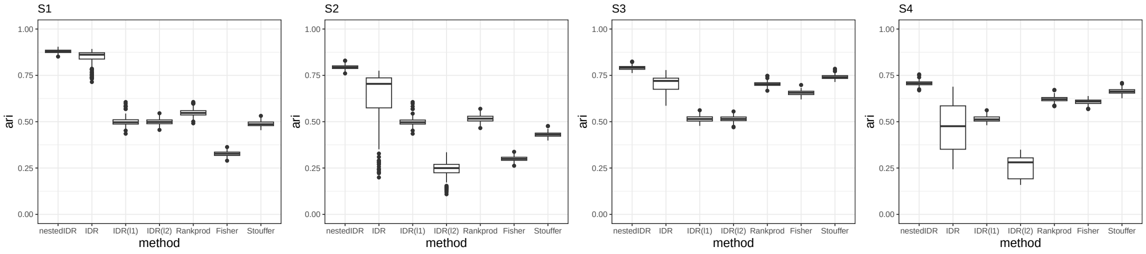

(b) ARI

Supplementary Figure 2: Comparison of five methods (nestedIDR, IDR, RankProd, Fisher, and Stouffer) when irreproducible genuine signals within a lab are dependent ($\rho_{0|1}$ =0.2). These are evaluated across four simulation scenarios (S1* to S4*). IDR: IDR applied to all replicates; IDR(l1) and IDR(l2): IDR applied to replicates in lab 1 and lab 2, respectively. (a) ROC curve (b) Adjusted rand index (ARI).

where $x_i^{(1)}$ and $x_i^{(2)}$ denote the measurements from the first and second lab, respectively. This transformation was applied to all four simulation scenarios (S1–S4) described in the main text.

Supplemental Figure 3a shows that nestedIDR maintains consistently higher true positive rates than all competing methods, including IDR, RankProd, Fisher, and Stouffer. Supplemental Figure 3b further demonstrates that nestedIDR achieves the highest classification accuracy, as measured by the Adjusted Rand Index (ARI), across all scenarios. These results indicate that nestedIDR remains robust to moderate violations of the cross-lab independence assumption and is well-suited for analyzing data from heterogeneous multi-lab settings.

3.4 Simulations for evaluating the robustness of nestIDR to misspecified copula family

To evaluate the robustness of nestedIDR to misspecified dependence structure among reproducible signals, we generated the reproducible components ($k = 1 \mid g = 1$) from a Gumbel copula with dependence parameter $\theta = 2$ instead of the Gaussian copula assumed by nestedIDR.

Supplemental Figure 4a shows that nestedIDR maintains consistently higher true positive rates than all competing methods, including IDR, RankProd, Fisher, and Stouffer. Supplemental Figure 4b further demonstrates that nestedIDR achieves the highest classification accuracy, as measured by the Adjusted Rand Index (ARI), across all scenarios. These results indicate that nestedIDR remains robust to moderate misspecification of the dependence assumption of reproducible components.

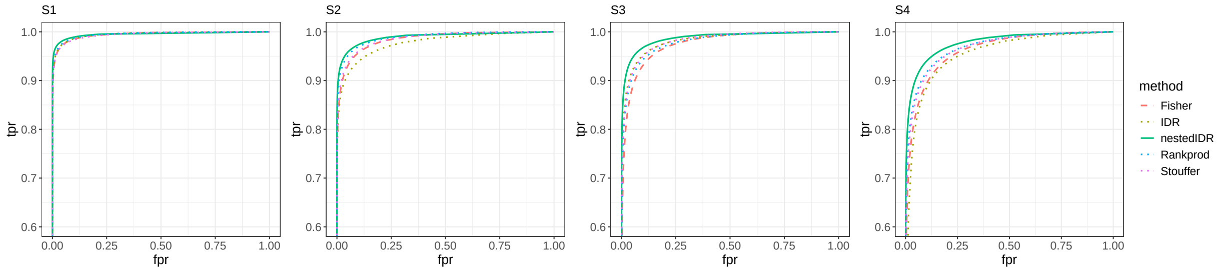

(a) ROC curves

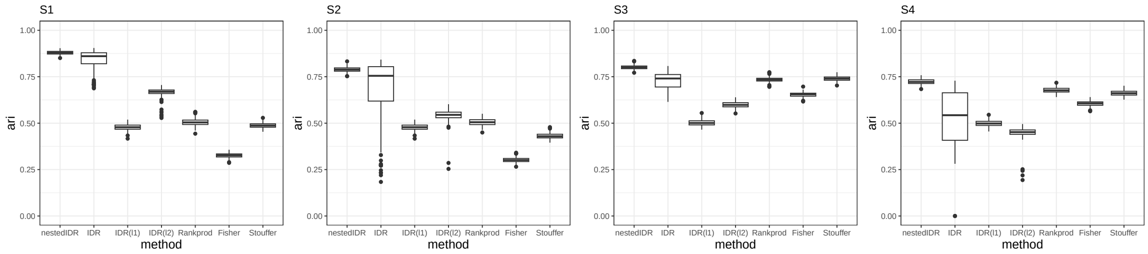

(b) ARI

Supplementary Figure 3: Performance comparison of nestedIDR, IDR, RankProd, Fisher, and Stouffer under mild cross-lab correlation. The four scenarios follow the same settings as the original S1-S4 simulations, with cross-lab dependence introduced via $x_i^{(2)} = x_i^{(2)} + 0.2x_i^{(1)}$, where $x_i^{(1)}$ and $x_i^{(2)}$ are measurements from labs 1 and 2, respectively. (a) ROC curves (b) Adjusted rand index (ARI) for classifying replicability of signals. IDR: IDR applied to all replicates; IDR(l1) and IDR(l2): IDR applied to replicates in lab 1 and lab 2, respectively.

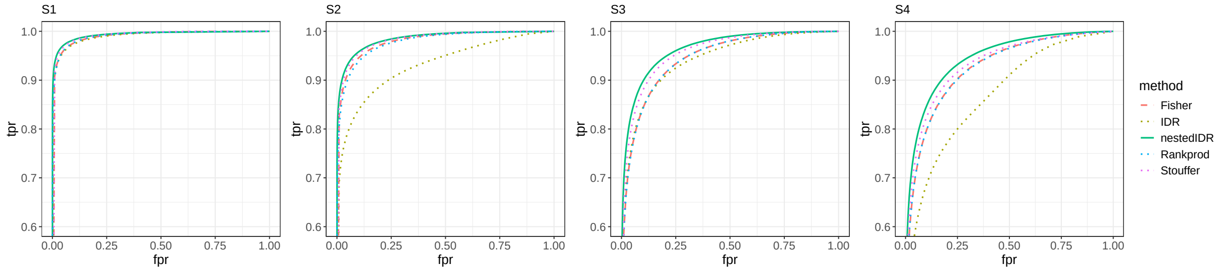

(a) ROC curves

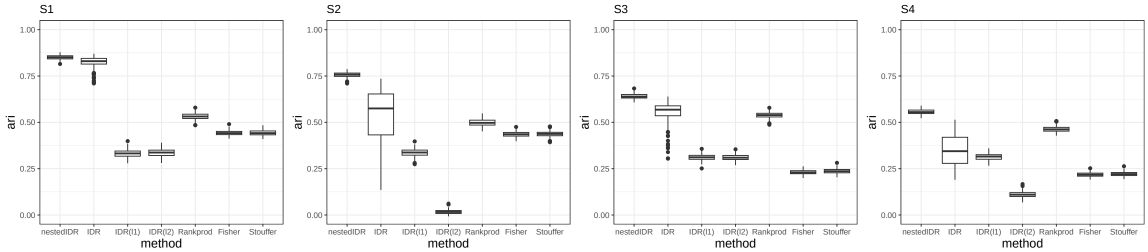

(b) ARI

Supplementary Figure 4: Performance comparison of nestedIDR, IDR, RankProd, Fisher, and Stouffer when the dependence of reproducible components ($k = 1 \mid g = 1$) between replicates within a lab is generated from a Gumbel copula instead of the Gaussian copula assumed by nestedIDR. The four scenarios follow the same settings as the original S1-S4 simulations, with dependence introduced via a Gumbel copula with dependence parameter $\theta = 2$. (a) ROC curves (b) Adjusted rand index (ARI) for classifying replicability of signals. IDR: IDR applied to all replicates; IDR(l1) and IDR(l2): IDR applied to replicates in lab 1 and lab 2, respectively.
